# Worse In-Hospital Outcomes Among Patients With Heart Failure (HF) and Concomitant Influenza Infection

**DOI:** 10.7759/cureus.32925

**Published:** 2022-12-25

**Authors:** Andrea Torres, Anoj Shahi, Karol Quelal, Saurabh Malhotra

**Affiliations:** 1 Cardiology, John H. Stroger, Jr. Hospital of Cook County, Chicago, USA; 2 Internal Medicine, John H. Stroger, Jr. Hospital of Cook County, Chicago, USA

**Keywords:** hospital outcomes, influenza, diastolic heart failure, systolic heart failure, heart failure

## Abstract

Introduction: A sizable proportion of heart failure (HF) admissions is precipitated by respiratory infections. Influenza has been linked to higher rates of HF hospitalizations and in-hospital morbidity and mortality.

Aim/Objective: We aim to describe the in-hospital outcomes of systolic HF vs. diastolic HF admissions with concomitant influenza infection in US hospitalizations from 2016 to 2017.

Materials and Methods: We queried the National Inpatient Sample (NIS) from 2016 to 2017 for discharge diagnosis for SHF and DHF and influenza per ICD-10 CM codes. Using binominal logistic regression analysis and adjusting for demographic and comorbid conditions, we compared the outcomes of SHF vs. DHF admissions with concomitant influenza as an independent risk factor for inpatient mortality, acute respiratory failure, ICU admission, assisted ventilation, as well as length of stay, and total hospital costs.

Results: A total of 7,490,596 HF weighted admissions were analyzed, among which 0.9% had concomitant influenza infection. SHF and DHF admissions with influenza had higher mortality, ICU admission, ventilation assistance, and acute respiratory failure when compared to those without influenza. Among influenza admissions, those with SHF had higher mortality (6.6% vs. 5%, adjusted odds ratio - aOR 1.31, p<0.001) compared to DHF. While intensive care unit (ICU) admission (7.8% vs. 5.2%, aOR 1.30, p<0.001) and ventilation assistance rates (22.1% vs. 18.9%, aOR 1.15, p<0.001) were greater among SHF patients with influenza, acute respiratory failure was more common amongst diastolic HF with influenza (46.6% vs. 51.2%, aOR 0.86, p<0.001). Finally, SHF patients with concomitant influenza had higher inpatient costs ($82,788) when compared to diastolic HF patients ($66,373) and a longer in-hospital stay (7.29 days compared to 6.98 days in the diastolic HF group) p <0.001.

Conclusion: Concomitant influenza infection in hospitalized patients with HF is associated with higher mortality, ICU admission, and the need for assisted ventilation, especially in those with SHF. A greater emphasis on vaccination against influenza may improve in-patient outcomes among HF patients.

## Introduction

Heart failure (HF) affects approximately 26 million people worldwide [1] and is projected to affect more than 8 million adults in the United States by 2030 [2]. In 2017 alone, HF was reported as the underlying cause of 80,480 deaths in the United States [3]. Similarly, seasonal influenza results in 3-5 million cases of severe illness and approximately 650,000 annual deaths worldwide [4]. In the United States, influenza was responsible for about half a million hospitalizations from 2016 to 2017 [5]. As a result, influenza puts an undue burden on the healthcare system, with an estimated median annual cost of $11.2 billion [6]. An epidemiologic study from Olmsted County, Minnesota, in 2000 to 2010, attributed 54% of HF deaths and 63% of HF hospitalizations to non-cardiovascular causes [7]. Respiratory infections, specifically, may precipitate up to 15.3% of HF admissions and increase in-hospital mortality by 60% [8]. Influenza has been linked to higher rates of HF hospitalizations and in-hospital mortality [9], although little has been described regarding influenza effects on systolic heart failure (SHF) vs diastolic heart failure (DHF). Hence, in this analysis from the National Inpatient Sample (NIS), we aimed to determine the differences in clinical outcomes among SHF vs DHF admissions with concomitant influenza in US hospitalizations from 2016 to 2017.

## Materials and methods

This is a retrospective analysis of HF admissions with influenza between 2016 and 2017 identified in the NIS as discharge diagnosis by the International Classification of Diseases, Tenth Revision, Clinical Modification (ICD-10 CM). We classified HF as systolic (I502X) and diastolic (I503X), codes that have previously correlated to HF with reduced ejection fraction (EF) [10] and HF with preserved EF [11], respectively. We excluded right ventricular, bi-ventricular, and unspecified HF from this analysis to facilitate the comparison between SHF and DHF. The table in the Appendix describes the different ICD-10 CM codes used and their specifications.

The NIS is part of the Healthcare Cost and Utilization Project (HCUP), a family of healthcare databases and related software tools and products developed through a federal/state/industry partnership sponsored by the Agency for Healthcare Research and Quality. The NIS is the largest publicly available all-payer inpatient healthcare database in the United States, yielding national estimates of hospital inpatient stays. It consists of a stratified systematic sample of discharges from all hospitals in HCUP, equal to approximately 20% of all discharges in US community hospitals. When weighed, it estimates more than 35 million hospitalizations per year nationally.

Influenza was analyzed as an independent risk factor for in-hospital outcomes using binominal logistic regression analysis and adjusting for demographic and comorbid conditions. The outcomes assessed were: 1) in-patient mortality, 2) acute respiratory failure (ARF), 3) admission to the intensive care unit (ICU), and, 4) need for assisted ventilation. We identified ARF per ICD-10 CM codes, which encompasses acute and acute on chronic respiratory failure with and without hypoxia and hypercapnia. Also we identified the need for assisted ventilation per ICD-10 PCS codes which includes mechanical and non-mechanical ventilation along with other respiratory support systems such as high flow nasal cannula. The ICU variable was created using surrogate conditions that require ICU care, such as continuous invasive mechanical ventilation for over 96 h, arterial catheterization, systemic arterial pressure monitoring, or central venous pressure monitoring (table in the Appendix). This approach has been used in prior studies from the NIS database [12]. Differences in length of stay and hospital costs were assessed using variables provided within the NIS. Discharge weights generated national estimates as recommended by the Agency for Healthcare Research and Quality. Chi-square and Mann-Whitney U tests measured statistical differences between categorical and continuous variables, respectively. A p-value of <0.05 was considered statistically significant. All statistical analyses were performed using the Statistical Package for the Social Sciences (SPSS Statistics version 22.0 - IBM Corp., Armonk, NY).

## Results

A total of 7,490,596 weighted HF admissions were analyzed, of which, 3,827,743 (51.5%) had SHF and 3,631,833 (48.5%) had DHF. Among the total HF admissions, 67,780 (0.9%) had concomitant influenza while hospitalized (51.7% with SHF and 48.5% with DHF).

When compared to SHF, the majority of admissions were women (60% vs. 38.4%, p=<0.001), had a greater prevalence of valvular heart disease (7.7% vs. 5.9%, p=<0.001), chronic pulmonary disease (35.3% vs. 29.4%, p=<0.001), diabetes (40.9% vs. 39.4%, p=<0.001), obesity (23.5% vs. 15.8%, p=<0.001) and hypertension (58.6% vs. 50.3%, p=<0.001). Although there were statistical differences in liver disease (3.8% vs. 3.5%, p=<0.001), malignancy (4.9% vs. 4.5%, p=<0.001), and renal failure (36.5% vs. 36.8%, p=<0.001), these were marginal.

Within the SHF subgroup, those with influenza were more often women (42% vs. 38.4%, p=<0.001) with a mean age of 72 ± 14 vs. 70 ± 14 (p=<0.001) compared to non-influenza admissions. Similarly, among DHF admissions, those with influenza were more often women (62.2% vs. 60%, p=<0.001) with a mean age of 76 ± 13 vs. 74 ± 13 years (p=<0.001) compared to non-influenza admissions. Table 1 describes the clinical characteristics of the stratified cohort by type of HF and influenza status. 

**Table 1:** Baseline characteristics of HF admissions stratified according to HF phenotype and influenza status. Baseline characteristics and comorbid conditions of HF admissions stratified according to HF phenotype and influenza status. The statistical differences represented in this table were measured by a Chi-square statistic. A p-value <0.05 was considered statistically significant. HF, heart failure; SHF, systolic heart failure; DHF, diastolic heart failure; SD, standard deviation

Characteristics	SHF			DHF		
	Without influenza	With influenza	p-value	Without influenza	With influenza	p-value
No. of observations (weighted)	3,845,008 (51.3%)	31,190 (0.4%)	-	3,595,073 (47.8%)	36,760 (0.5%)	-
Female sex	38.4%	42%	< 0.001	60%	62.2%	< 0.001
Age (mean ± SD)	70 ± 14	72 ± 14	< 0.001	74 ± 13	76 ± 13	< 0.001
White	65.2%	64.2%	< 0.001	71%	69.9%	< 0.001
Non-White	34.8%	35.8%	< 0.001	29%	30.1%	< 0.001
Valvular disease	5.9%	9%	< 0.001	7.7%	10.4%	< 0.001
Peripheral vascular disease	9.6%	6.8%	< 0.001	8.9%	7.3%	< 0.001
Chronic pulmonary disease	29.4%	38.4%	< 0.001	35.3%	44.3%	< 0.001
Diabetes	39.5%	37.8%	< 0.001	41%	37.4%	< 0.001
Renal failure	36.9%	35.2%	< 0.001	36.6%	33%	< 0.001
Liver disease	3.5%	2.5%	< 0.001	3.8%	2.5%	< 0.001
Malignancy	4.5%	4.5%	0.86	4.9%	4.5%	0.001
Obesity	15.8%	14.4%	< 0.001	23.5%	21.3%	< 0.001
Hypertension	50.3%	61.6%	< 0.001	58.5%	70.3%	< 0.001
Substance use	6.1%	4.7%	< 0.001	3.7%	3%	< 0.001

Influenza admissions had a greater prevalence of valvular disease, chronic pulmonary disease, and hypertension in both the SHF and DHF groups compared to admissions with no influenza. On the contrary, peripheral vascular disease, diabetes, renal failure, liver disease, and obesity were more prevalent in HF admissions without influenza (Table 1).

**Figure 1:**
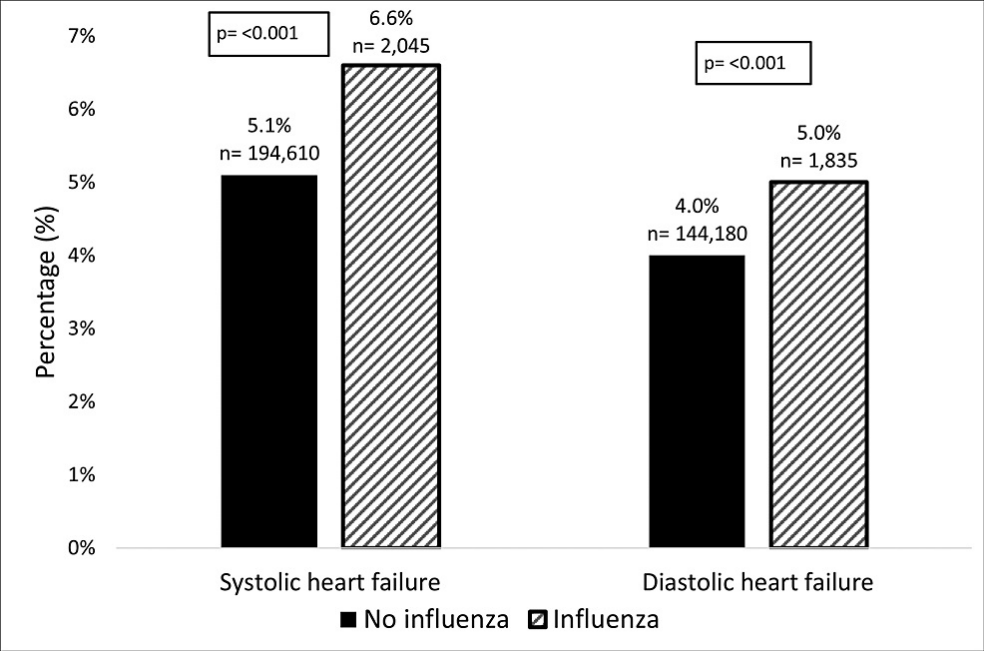
Mortality rate among HF admissions stratified by HF type and influenza status. n, number of observations; p, p-value for Chi-square test; HF, heart failure

Overall, HF admissions with influenza had higher inpatient mortality (5.7% vs. 4.6%, p<0.001), higher ICU admission rates (6.4% vs. 3.3%, p<0.001), and ARF (49.1% vs. 26.6%, p<0.001) when compared to those without influenza. Likewise, SHF with influenza had higher mortality (6.6% vs. 5.1%, p<0.001), ICU admission (7.8% vs. 3.7%, p<0.001), ventilation assistance (22.1% vs. 12.2%, p<0.001), and ARF (46.6% vs. 23.9%, p<0.001) than those without influenza. And DHF with influenza resulted in more deaths (5% vs. 4%, p<0.001), greater ICU admission (5.2% vs. 2.9%, p<0.001), ventilation assistance (19% vs. 12.3%, p<0.001) and ARF (51.2% vs. 29.4%, p<0.001) when compared to those without influenza (Figure 1). 

**Figure 2:**
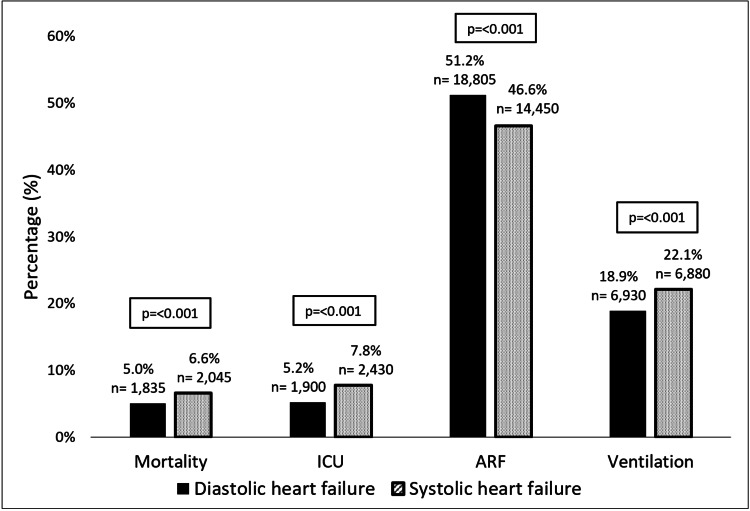
Outcomes among admissions with SHF and DHF and concomitant influenza infection. Outcome comparison between SHF and DHF admissions with concomitant influenza. ARF, acute respiratory failure; ICU, intensive care unit; n, number of observations; p, p value for Chi square test ICU admission rate inferred by surrogate conditions that require an ICU setting (continuous invasive mechanical ventilation >96 h, arterial catheterization, systemic arterial pressure monitoring, or central venous pressure monitoring). Ventilation includes assistance by mechanical, non-invasive ventilation and other respiratory support systems such as high flow nasal cannula.

Among the influenza cohort, SHF had more deaths (6.6% vs. 5% respectively, p<0.001) and ICU admissions (7.8% vs. 5.2%, p<0.001) compared to DHF. On the contrary, ARF was more common amongst DHF with influenza compared to SHF with influenza (46.6% vs. 51.2%, p<0.001), although SHF still showed higher rates of assisted ventilation (22.1% vs. 18.9%, p<0.001) (Figure 2). 

The effect of HF and concomitant influenza on outcomes was ascertained by multivariate logistic regression analysis using DHF without influenza as the comparison group. After adjusting for demographic and other comorbid conditions, SHF with influenza had the highest odds of mortality (aOR 1.69; 95% CI: 1.61-1.77; P<0.001), ICU admission (aOR 2.58, CI 2.47-2.69; p<0.001), and need for assisted ventilation (aOR 1.97, CI 1.91-2.02; p<0.001) within the analyzed groups (Table 2). However, DHF patients with influenza had greater odds for ARF (aOR 2.44, CI 2.39-2.50, p<0.001). A head-to-head comparison of SHF with influenza and DHF with influenza can be found in the supplementary material. 

**Table 2:** aOR by subgroups of HF and influenza in a multivariate regression model (as compared to admissions with DHF without influenza). aOR for the outcomes of mortality, ICU admission, acute respiratory failure, and assisted ventilation as compared to admissions with diastolic heart failure without influenza who were presumed to be the group with the lowest risk. *The presented odds ratios were adjusted to demographic variables (age, sex, and race) and by the comorbidities listed in Table 1. HF, heart failure; aOR, adjusted odds ratio; DHF, diastolic heart failure; CI, confidence interval

Outcome	DHF influenza negative (reference group)	DHF influenza positive aOR* (95% CI)	p-value	SHF influenza negative aOR (95% CI)	p-value	SHF influenza positive aOR (95% CI)	p-value
Mortality	-	1.25 (1.19-1.31)	<0.001	1.29 (1.28-1.30)	<0.001	1.69 (1.61-1.77)	<0.001
ICU admission	-	1.93 (1.85-2.03)	<0.001	1.09 (1.08-1.10)	<0.001	2.58 (2.47-2.69)	<0.001
Acute respiratory failure	-	2.44 (2.39-2.50)	<0.001	0.81 (0.80-0.81)	<0.001	2.17 (2.12-2.22)	<0.001
Assisted ventilation	-	1.63 (1.58-1.67)	<0.001	0.98 (0.97-0.98)	<0.001	1.97 (1.91-2.02)	<0.001

Finally, HF admissions with influenza had higher inpatient costs ($75,029 vs. $71,184; p<0.001) and longer lengths of stay (7.16 days vs. 6.40 days; p<0.001) compared to those without influenza. Furthermore, SHF with influenza showed higher costs of care ($82,788 vs. $66,373; p<0.001) and a longer in-hospital stay (7.29 days vs. 6.98 days; p<0.001) when compared to DHF with influenza.

## Discussion

There is ample evidence of the detrimental effects of influenza in patients with cardiovascular disease either by causing viral myocarditis [13-14], creating a pro-inflammatory state [15-16], or causing microcirculatory abnormalities [17]. Furthermore, influenza poses a severe risk of dehydration which can further deteriorate renal function in patients with pre-existing heart conditions using diuretics and renin-angiotensin-aldosterone system inhibitors. This links with findings from a similar NIS study performed with data between 2013 and 2014 [9] as well as our own. Both analyses showed increased in-patient mortality among HF patients with influenza. Although this prior study did not differentiate between outcomes in SHF and DHF.

In this analysis, we observed higher mortality, ICU admission, and assisted ventilation rates in SHF compared to DHF within the influenza cohort. Historically, SHF has demonstrated increased mortality compared to DHF [18-19], along with significant differences in its mortality predictors [20]. This could be explained by a poor contractile reserve in SHF and, therefore, a higher risk for vasopressor support and ICU admission, especially when encountering the extra burden of a systemic illness such as influenza. Moreover, others have postulated that the inability to optimally dialyze SHF patients with end-stage renal disease (ESRD) due to fragile hemodynamics can contribute to increased mortality in this group [12].

Likewise, ARF was more common among HF admissions with influenza (49.1% vs. 26.6%, p<0.001) compared to those without influenza, similar to findings by Panhwar et al. [9]. However, on subgroup analysis within our influenza cohort, DHF was more likely to have ARF when compared to SHF (51.1% vs. 46.7%, p<0.001). This difference could be accounted for by a greater prevalence of lung function abnormalities in DHF patients [21]. As noted in our cohort, chronic obstructive pulmonary disease (COPD) (44.4% vs. 38.4%, p=<0.001) and obesity (21.3% vs. 14.4%, p=<0.001) were more common among DHF admissions. Obesity poses mechanical barriers to proper ventilation and is associated with a high prevalence of obstructive sleep apnea (51.5%) [22] and obesity hypoventilation syndrome (31%) in patients with a body mass index (BMI) ≥35 [23], which further increases the risk for ARF and the need for ventilatory support, especially in the setting of respiratory infections [24]. Similarly, obesity is known to cause a chronic inflammatory state, even within the airways, through the release of IL-6 from adipocytes and facilitating the release of other pro-inflammatory cytokines and the generation of reactive oxygen species [25]. Obesity is thus potentially linked to increased incidence of respiratory failure and ventilator needs among DHF patients. 

Finally, we found that SHF admissions with concomitant influenza had higher in-patient costs ($82,788 vs. $66,373, p<0.001) and longer in-hospital stays (7.29 days vs. 6.98 days, p<0.001) compared to DHF patients with influenza. This could partly be explained by the greater likelihood of ICU stay and a greater need for assisted mechanical ventilation among those with SHF and concomitant influenza infection.

Some studies show the beneficial outcomes of influenza vaccinations among HF patients [26-28]. Vardeny et al. conducted an observational study on the cohort of the PARADIGM-HF trial assessing influenza vaccination and outcomes in HF patients and found lower mortality among vaccinated HF patients [26]. However, this study could not determine causality because of its observational design. An observational study from nationwide registries conducted in Denmark showed an overall reduction in mortality by around 18% in HF patients receiving at least one influenza vaccine after HF diagnosis and 20% with annual vaccination frequency [27]. Despite the growing literature on improved cardiovascular outcomes with influenza vaccination [27-28], the major hindrance appears to be vaccination rates. During the influenza season of 2016-2017, the Centers for Disease Control and Prevention (CDC) reported a vaccination coverage of only 43.3% on the adult population with a marginal increase to 48.4% in 2019-2020 [29]. Putting this into perspective, with an estimated HF prevalence of 6.2 million Americans ≥20 years of age based on data from the National Health and Nutrition Examination Survey (NHANES) from 2013 to 2016 [3], if we assume the same vaccination coverage as the general population during these years, more than two million adults with HF are at risk of higher mortality and poor in-patient outcomes. Moreover, a quality improvement initiative by American Heart Association using the Get With The Guidelines-Heart Failure registry showed that one in three patients admitted for HF were not vaccinated for influenza [30]. Influenza vaccination is a simple yet effective tool at our disposal that, regardless of the HF type, can be effective in improving the prognosis of HF patients with concomitant influenza infection. 

Limitations 

The NIS is based on administrative data created mainly to facilitate billing purposes and lacks clinical details. As such, the ICD-10 codes for the clinical conditions described in this study heavily rely on the judgment of the person entering the codes during the hospital stay, including the classification between SHF and DHF. The number of publications validating the use of ICD-10 codes to accurately identify SHF and DHF is limited, with a reported sensitivity between 68% and 72% for SHF and 35% and 46% for DHF, and a specificity between 63% and 68% for SHF and 84% and 91% for DHF [11-12]. Hence, the included cases in each group might underestimate their real prevalence, especially in the SHF population.

Furthermore, the comparison groups in this analysis consist of a small HF with influenza group compared to a significantly larger HF without influenza group, which is likely to facilitate statistically significant results even though the differences may be small. Since NIS is mainly based on inpatient records, our results cannot be accurately extrapolated in the outpatient setting. Thus, extrapolating these results into clinical practice should be taken with caution.

It should also be noted that information on influenza vaccination status is not available on the NIS, and differences in outcomes among HF patients who are vaccinated vs. those who are not cannot be ascertained. Additionally, the severity of concomitant influenza infection and its management (for example, the use of Oseltamivir) is not known and thus cannot be studied as a predictor of outcomes.

## Conclusions

Concomitant influenza infection in hospitalized patients with HF is associated with significant morbidity and mortality. Our study reports higher mortality in the SHF population along with higher inpatient costs and length of stay compared to diastolic HF. Diastolic HF patients with concomitant influenza infection are more likely to develop acute respiratory failure when compared to those with SHF and concomitant influenza infection. Vaccination efforts targeted at HF patients are warranted to reduce the risk of detrimental hospital outcomes in this population. 

## Appendices

**Table 3:** Outcomes in influenza admissions with SHF vs DHF. Head-to-head comparison between SHF with influenza and DHF with influenza HF, heart failure; aOR, adjusted odds ratio; CI, confidence interval; SHF, systolic heart failure; DHF, diastolic heart failure *The presented odds ratios were adjusted to demographic variables (age, sex, and race) and by the comorbidities listed in Table 1

Outcome	SHF with influenza	DHF with influenza	aOR* (95% CI)	p-value
	N (%)	N (%)		
Mortality	2055 (6.6%)	1825 (5%)	1.24 (1.16-1.32)	<0.001
ICU admission	2435 (7.8%)	1895 (5.2%)	1.44 (1.35-1.54)	<0.001
Acute respiratory failure	14540 (46.6%)	18715 (51.1%)	0.88 (0.85-0.91)	<0.001
Assisted ventilation	6880 (22.1%)	6930 (18.9%)	1.21 (1.17-1.26)	<0.001

**Table 4:** ICD-10 CM codes and ICD-10 PCS codes. Codification system used to identify discharge diagnoses registered in the NIS database. The clinical codes are registered as ICD-10 CM and the procedural codes as ICD-10 PCS such as those used for assisted ventilation. SHF, systolic heart failure; DHF, diastolic heart failure; NIS, National Inpatient Sample

Disease	ICD-10 CM code	Description
SHF	I5020	Unspecified systolic (congestive) heart failure
	I5021	Acute systolic (congestive) heart failure
	I5022	Chronic systolic (congestive) heart failure
	I5023	Acute on chronic systolic (congestive) heart failure
	I5040	Unsp combined systolic and diastolic (congestive) hrt fail
	I5041	Acute combined systolic and diastolic (congestive) hrt fail
	I5042	Chronic combined systolic and diastolic hrt fail
	I5043	Acute on chronic combined systolic and diastolic hrt fail
DHF	I5030	Unspecified diastolic (congestive) heart failure
	I5031	Acute diastolic (congestive) heart failure
	I5032	Chronic diastolic (congestive) heart failure
	I5033	Acute on chronic diastolic (congestive) heart failure
Influenza	J09X1, J09X2, J09X3, J09X9, J1000, J1001, J1008, J101, J102, J1081, J1082, J1083, J1089, J1100, J1108, J111, J112, J1181, J1182, J1183, J1189	Influenza due to different identified and unidentified influenza viruses with and without complications
Acute respiratory failure	J9600	Acute respiratory failure, unsp w hypoxia or hypercapnia
	J9601	Acute respiratory failure with hypoxia
	J9602	Acute respiratory failure with hypercapnia
	J9690	Respiratory failure, unsp, unsp w hypoxia or hypercapnia
	J9691	Respiratory failure, unspecified with hypoxia
	J9692	Respiratory failure, unspecified with hypercapnia
	J9620	Acute and chr resp failure, unsp w hypoxia or hypercapnia
	J9621	Acute and chronic respiratory failure with hypoxia
	J9622	Acute and chronic respiratory failure with hypercapnia
ICU admission		
Continuous mechanical ventilation	5A1955Z	Respiratory ventilation, greater than 96 consecutive hours
Arterial catheterization	03HY32Z, 03HY33Z, 03HY42Z, 03HY43Z, 04HY32Z, 04HY33Z, 04HY42Z, 04HY43Z	Insertion of monitoring or infusion device into artery, percutaneous approach
Systemic arterial pressure monitoring	4A130B1, 4A133B1, 4A13XB1	Monitoring of arterial pressure, peripheral, open, percutaneous, and external approach
Central venous pressure monitoring	4A140B0, 4A143B0	Monitoring of venous pressure, central, open, and percutaneous approach
Assisted ventilation	5A09357, 5A09358, 5A09359, 5A0935A, 5A0935B, 5A0935Z, 5A09457, 5A09458, 5A09459, 5A0945A, 5A0945B, 5A0945Z, 5A09557, 5A09558, 5A09559, 5A0955A, 5A0955B, 5A0955Z, 5A19054	Assistance with respiratory ventilation, <24 h, 24-96 h or >96 h, with either CPAP, intermittent positive air, continuous negative air, high nasal flow vel, intermittent negative air. OR respiratory ventilation, single, non-mechanical
Mechanical ventilation	5A1935Z, 5A1945Z, 5A1955Z	Respiratory ventilation, less than 24 consecutive hours, 24-96 consecutive hours, OR greater than 96 consecutive hours
